# Network pharmacology, machine learning, and experiments uncover β-sitosterol targeting HSP90AA1 in medicinal-edible black soybean against aging

**DOI:** 10.3389/fmed.2026.1749856

**Published:** 2026-02-16

**Authors:** Yuanyuan Jia, Jingyi Yang, Qian Chen, Yuting Yang, Wei Min, Dan Luo

**Affiliations:** 1 Department of Dermatology, The First Affiliated Hospital with Nanjing Medical University, Nanjing, Jiangsu, China; 2 Department of Dermatology, Kunshan First People’s Hospital, Suzhou, Jiangsu, China

**Keywords:** aging, black soybean, HSP90AA1, machine learning, network pharmacology, β-sitosterol

## Abstract

**Background:**

Aging involves progressive dysregulation of cellular homeostasis, while current anti-aging agents face limitations in specificity and safety. Black soybean, a medicinal-edible substance, contains various bioactive compounds. This study aims to investigate the anti-aging mechanism of its key component, β-sitosterol, through integrated network pharmacology and machine learning.

**Methods:**

Active components and targets of black soybean were acquired from the TCMSP database, while aging-associated genes were obtained from the GeneCards, TTD, and OMIM databases. Venn analysis was applied to intersect the targets. A protein-protein interaction (PPI) network was constructed using the STRING database and visualized with Cytoscape software to identify core targets. Gene Ontology (GO) and Kyoto Encyclopedia of Genes and Genomes (KEGG) analyses were conducted via Metascape. A triple-machine-learning strategy (SVM-RFE, RF, LASSO) was employed to further prioritize key targets. Molecular docking was performed to predict the binding affinities. The core targets and mechanisms were validated through *in vitro* assays in a UVA-induced human skin fibroblast (HSF) model.

**Results:**

Bioinformatics analysis identified 63 overlapping targets between the corresponding targets of black soybean components and aging-associated genes, with protein-protein interaction networks prioritizing HSP90AA1 and BCL2 as core regulators. Molecular docking confirmed β-sitosterol’s high-affinity binding to HSP90AA1, primarily through hydrogen bond interactions with the key amino acid residue ILE-214 within its 9-236 region, which is the functional domain responsible for client protein interaction. Experimental validation in cellular models demonstrated β-sitosterol attenuated photoaging markers, restored cell cycle arrest, and enhanced antioxidant defenses. Mechanistically, β-sitosterol upregulated HSP90AA1 expression to stabilize apoptotic regulators (BCL2, p53) and mitigate oxidative damage. Inhibition of HSP90AA1 abolished these effects, establishing its pivotal role.

**Conclusion:**

This work reveals that β-sitosterol, a core component of black soybean, combats skin photoaging by targeting the HSP90AA1-mediated stress adaptation and regulating the p53-BCL2 signaling axis. These findings provide a molecular basis for the application of this medicinal-edible substance in anti-aging interventions.

## 1 Introduction

Aging is a complex biological process coordinately controlled by genetic networks, signaling pathways, and molecular mechanisms. Essentially, aging is a progressive imbalance of cellular homeostasis, involving multi-dimensional mechanisms such as oxidative stress, protein homeostasis dysregulation, and epigenetic alterations (1). Although anti-aging drugs like rapamycin and senolytics have entered the clinical trial stage, they suffer from insufficient target specificity and long-term toxicity, which severely limit their clinical applications (2). While traditional Chinese medicine (TCM) components that are both edible and medicinal, featuring multitarget regulation and good safety, have attracted extensive attention. These components are rich in bioactive compounds with relatively low toxicity.

Black soybean (*Glycine max* L.), recorded as a tonic in Compendium of Materia Medica, harbors a synergistic antioxidant network comprising polyphenols, isoflavones, and trace minerals (3). This phytochemical ensemble operates through complementary mechanisms: polyphenols neutralize reactive oxygen species, lipid-soluble antioxidants stabilize cellular membranes, and micronutrients enhance endogenous enzymatic defenses (4, 5). Such multi-target interactions underscore its potential value in studying plant-derived anti-photoaging agents. β-sitosterol, the most abundant phytosterol, has been proven to possess physiological functions such as regulating cholesterol metabolism, inhibiting inflammatory responses, and activating autophagy (6, 7). Notably, recent studies have found that β-sitosterol can influence cellular stress responses by acting on the molecular chaperone system. However, its specific mechanism in aging regulation remains unclear (8). Meanwhile, Heat Shock Protein 90 Alpha Family Class A Member 1 (HSP90AA1), a key regulator in maintaining protein folding homeostasis, has been found to be closely associated with the cellular aging process. It exerts anti-aging effects by promoting the expression of autophagy-related genes and can also upregulate the expression of the senescence-associated secretory phenotype (SASP) (9, 10). This dual effect makes HSP90AA1 a potential target for anti-aging drug development. However, a direct mechanistic link between β-sitosterol and the HSP90AA1-mediated stress adaptation pathway in the context of skin aging has not been established. This knowledge gap presents a critical opportunity to elucidate the precise molecular basis of black soybean’s anti-aging efficacy.

Network pharmacology provides a new paradigm for revealing the multitarget action essence of TCM by constructing a component-target-pathway network (11). The construction of a protein-protein interaction (PPI) network can systematically uncover the potential associations between drug components and disease targets.

However, aging is an inherently heterogeneous biological process. Different tissues and organs may undergo aging through different mechanisms. Skin aging has drawn significant attention due to its impact on external appearance and quality of life. Skin serves as an ideal model for aging research due to its accessibility. Exogenous skin aging is mainly caused by environmental factors such as ultraviolet (UV) radiation, a process known as photoaging (12). UV radiation triggers collagen degradation via matrix metalloproteinases (MMPs) activation, culminating in wrinkle formation, while simultaneously inducing DNA damage that compromises cellular repair mechanisms (13). This dual-pathway assault not only accelerates visible skin aging but also potentiates malignant transformation, particularly elevating risks of squamous cell carcinoma and basal cell carcinoma (14). Traditional disease databases can provide information on general aging-related targets but lack specific target annotations for skin aging. This data gap leads to tissue-specific biases in target prediction based on network pharmacology. To address this bottleneck, machine learning algorithms can identify key targets from a vast amount of data through feature selection (15). Tools like limma play important roles in analyzing data such as gene expression to assist machine learning in target screening (16). In this study, a triple-machine-learning strategy, combined with PPI network topological parameters, was employed to preferentially screen core targets highly relevant to skin aging, thereby facilitating a better understanding of the key molecular mechanisms involved in skin aging and the anti-aging effects of black soybean.

Molecular docking technology can verify the binding feasibility between components and targets at the structural level. For example, the molecular docking of compounds from Tinospora cordifolia with AKT1 revealed a strong binding affinity, which provides the structural foundation for its anti-aging properties (17). The collaborative application of these technologies offers a systematic solution for researching the anti-aging mechanisms of traditional Chinese medicine.

**FIGURE 1:**
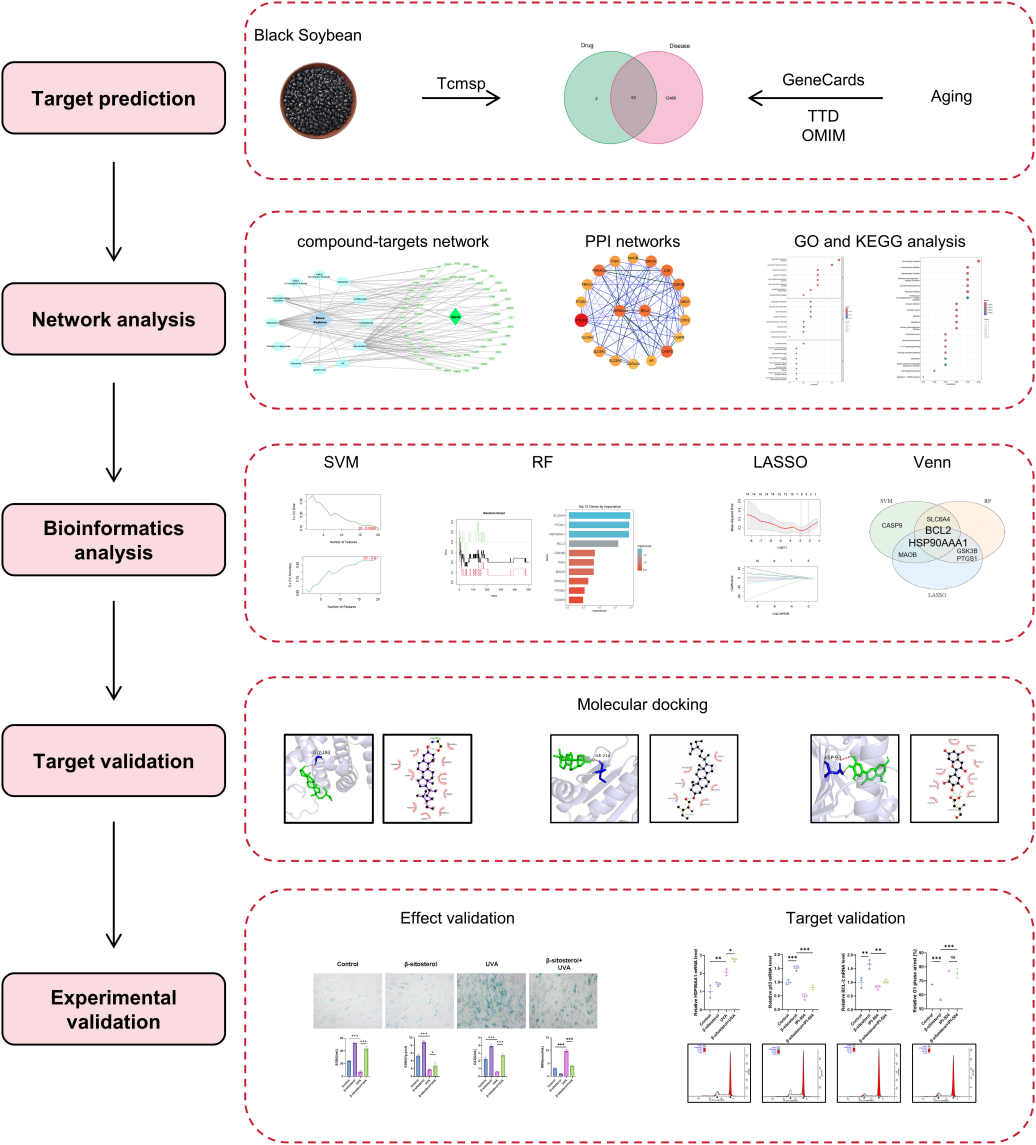
A flow-chart of this study.

This study integrated network pharmacology and machine learning to prioritize aging targets, identifying β-sitosterol as a core anti-aging component in black soybean. Molecular docking demonstrated its selective binding to HSP90AA1, a stress-response regulator, while *in vitro* assays confirmed its efficacy in mitigating photoaging. These findings demonstrate β-sitosterol’s dual role as a nutraceutical modulating HSP90AA1-mediated stress responses for skin aging intervention. The main scheme of this study is presented in Figure 1.

## 2 Materials and methods

### 2.1 Screening of active components and targets of black soybean

The Traditional Chinese Medicine Systems Pharmacology Database (TCMSP) was employed to retrieve the active components of black soybean and their corresponding targets. Prior to further analysis, we set screening criteria, requiring an oral bioavailability (OB) of no less than 30% and a drug-likeness (DL) of at least 0.18 (18). Subsequently, the main components of black soybean and their corresponding target proteins were identified. Using the Uniprot database, the gene names associated with these protein targets were successfully retrieved.

### 2.2 Identification of aging-related targets

**FIGURE 2:**
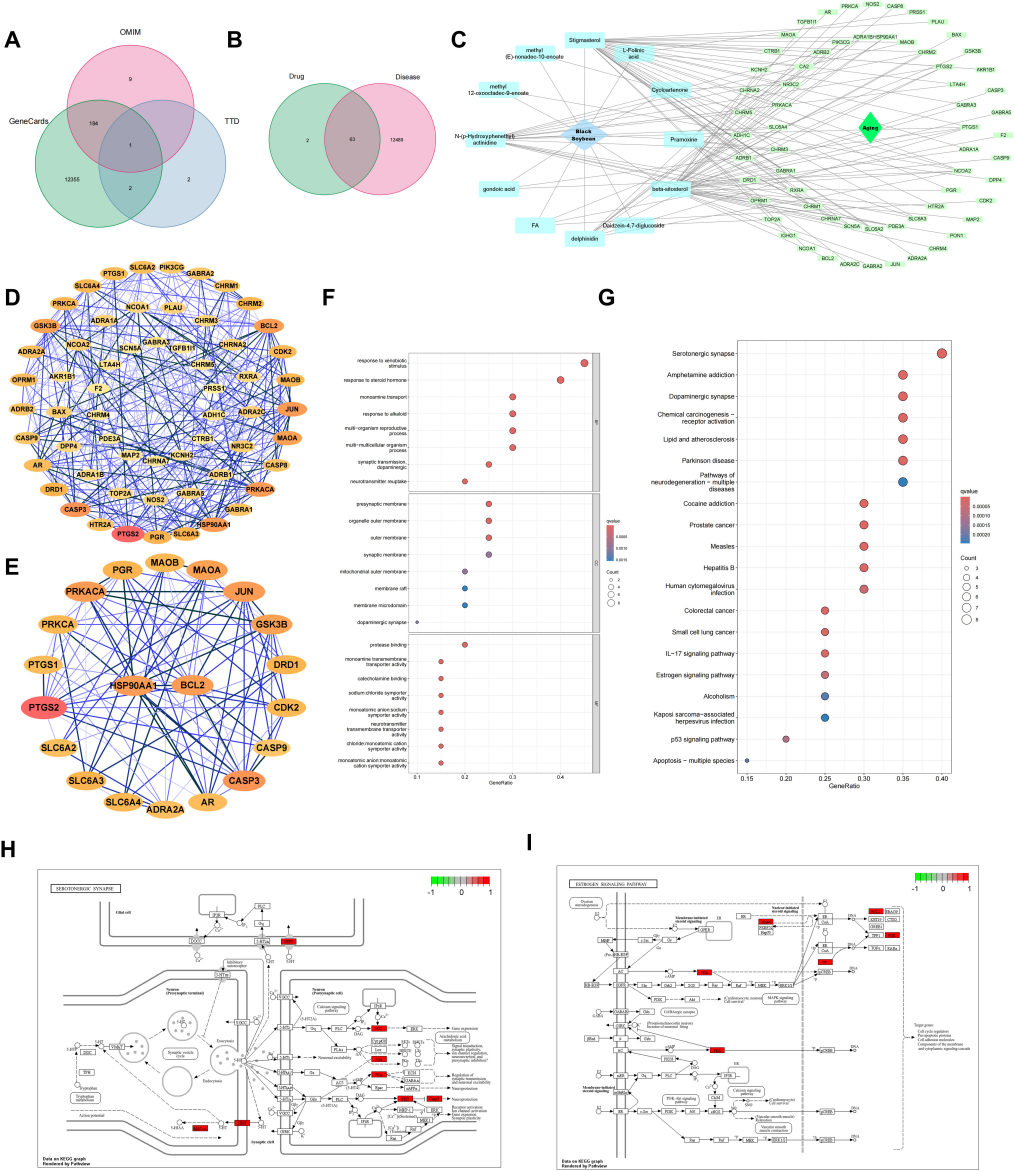
Network pharmacology. **(A)** Venn diagram showing the composition of aging-related targets retrieved from GeneCards, TTD, and OMIM databases. **(B)** Venn diagram identifying the overlapping targets between active components of black soybean and aging-related genes. **(C)** A compound-target network illustrating the connections between bioactive components of black soybean and the overlapping aging-related targets. **(D)** The PPI network of the overlapping targets, constructed using the STRING database and visualized in Cytoscape. Nodes represent proteins, edges represent interactions. **(E)** A detailed view of the core subnetwork extracted from **(D)**, highlighting topologically significant targets. Node size and color may reflect centrality metrics such as degree. **(F)** GO enrichment analysis results, displaying significantly enriched terms. **(G)** KEGG pathway enrichment analysis results, showing the top significantly enriched pathways. **(H,I)** Schematic diagrams of two key enriched pathways: **(H)** Serotonergic synapse and **(I)** Estrogen signaling pathway.

The GeneCards database, the TTD database, and the OMIM database were searched for aging-related disease targets (19, 20). By inputting the search term “aging,” targets related to this disease were retrieved from each database. Subsequently, the targets obtained from Genecards were screened using a standard relevance threshold of ≥ 1.0 to ensure the inclusion of genes with established evidence links to aging. The relative contributions of each database to the final target pool are visualized in Figure 2A. Then, the targets obtained from the databases were merged, and duplicate values were removed. This process ultimately determined the aging-related disease targets for further study.

### 2.3 Construction of the protein-protein interaction network

Meanwhile, the target data of black soybean active components and aging-related targets were imported into the JVENN website (21). A comprehensive compound-disease target network was constructed to elucidate the complex relationships between drug components and their corresponding disease targets. The intersection targets were uploaded to the STRING database to retrieve protein-protein interaction network information (22). Then, the PPI data were imported into the Cytoscape 3.10.0 software to visually present the complex interaction network.

### 2.4 Gene ontology enrichment analysis and Kyoto Encyclopedia of Genes and Genomes pathway analysis

Aging-related targets were analyzed via GO and KEGG enrichment using Metascape. GO terms covered biological processes (BP), cellular components (CC), and molecular functions (MF) (23, 24). In addition, KEGG identified critical signaling pathways in aging (25–28).

### 2.5 Machine learning

Skin tissue gene samples (GSE75192 from GEO) and PPI network data were analyzed using three algorithms: Support Vector Machine-Recursive Feature Elimination (SVM-RFE) (29), Random forest (RF) (30) and Least Absolute Shrinkage and Selection Operator (LASSO) (31). SVM-RFE: Gene selection was performed using a custom R script implementing the SVM-RFE algorithm. A linear kernel SVM was employed with a regularization parameter (cost) set to 10. The recursive feature elimination process used five-fold internal cross-validation to evaluate feature subsets. Features were ranked based on the squared weight vector of the linear SVM, and the least important features were sequentially eliminated. RF: The random forest model was constructed using the randomForest R package. An initial forest of 500 decision trees was grown, and the optimal number of trees (the point at which the out-of-bag error rate stabilized) was selected for the final model. Feature importance was calculated using the Gini index (mean decrease in impurity), and a threshold of > 0.25 was applied to prioritize genes. LASSO: LASSO logistic regression was implemented using the glmnet R package. A binomial family was specified for the binary classification task. The optimal regularization parameter (λ) was determined via five-fold cross-validation that minimized the mean squared error (MSE). A sequence of 100 λ values was tested to identify the model with the minimum cross-validation error. For all three algorithms, a robust validation strategy was employed: five-fold cross-validation was repeated three times to prevent overfitting and ensure the stability of the selected features. The random seeds were set to ensure reproducibility. When the results of these three machine-learning algorithms converged, the genes obtained from the intersection were identified as the key components for black soybean in treating skin aging.

### 2.6 Molecular docking

Black soybean compound structures (PubChem) were energy-minimized (Chem3D) (32). The three-dimensional structure of molecules were further optimized using the MMFF94 force field. Target protein structures were obtained from the RCSB Protein Data Bank and preprocessed by removing water molecules, adding hydrogen atoms, and assigning charges. Molecular docking simulations were performed using AutoDock Vina (version 1.1.2). The search algorithm employed was the Lamarckian Genetic Algorithm (LGA). A grid box centered on the protein’s active site with dimensions of 40 × 40 × 40 Å and a grid-point spacing of 0.375 Å was defined to allow for comprehensive ligand sampling. The binding affinity (ΔG, kcal/mol) was calculated using Vina’s default scoring function. The conformation with the most favorable binding energy was selected as the optimal pose. Optimal ligand-receptor conformations (highest affinity) were docked, visualized via PyMOL (33). And two-dimensional interaction diagrams were generated using LigPlus software.

### 2.7 Main reagents

We followed the materials and methods as we reported before (34). The SUV-100 solar simulator along with the radiant emittance monitor was sourced from Sigma Co., located in Shanghai, China. IPI-504 was acquired from ApexBio Technology in Houston, TX, United States. Tamoxifen was acquired from Sigma-Aldrich. A CCK-8 assay kit was bought from Beyotime Institute of Biotechnology in China. Assay kits for superoxide dismutase (SOD), glutathione peroxidase (GSH-Px), catalase (CAT), and malondialdehyde (MDA) were obtained from Nanjing Jiancheng Bioengineering Institute in Nanjing, China. A reverse transcription kit and a real-time quantitative PCR kit were purchased from TaKaRa Co., Dalian, China. The cytochemical staining kit for SA-β-gal was obtained from Mirus Bio Co., United States, and the ELISA kit for detecting extracellular Human Transforming Growth Factor-beta 1 (TGF-β1) was from R&D Co., United States. The ABI 7700 Real Time PCR System was from an Applied Biotechnology Company, United States.

### 2.8 Cell culture, β-sitosterol treatment, and UVA irradiation

Human skin fibroblasts (HSF) obtained from the Cell Center of Union Medical University were cultivated in Dulbecco’s Modified Eagle’s Medium (DMEM, supplied by Gibco/BRL, United States). The medium was supplemented with 10% heat-inactivated fetal bovine serum (FBS, procured from Invitrogen, United Kingdom), 100 U/mL penicillin, and 100 mg/L streptomycin. The cells were maintained at 37°C in an atmosphere containing 5% CO2. β-sitosterol (Tokyo Chemical Industry, Tokyo, Japan) was dissolved in the culture medium and used at a concentration of 25, 50 or 100 μg/mL for 24 h treatment. After treatment, the cells were sub-cultured when they reached half confluence (at a seeding density of 1 × 104 cells/cm^2^). Prior to exposure to UVA radiation, the cells were washed with phosphate-buffered saline (PBS). After the UVA irradiation of 10, 20 or 30 J/cm^2^, the culture medium was replaced with fresh medium.

### 2.9 Cell viability assay

The proliferation of HSF was evaluated according to the manufacturer’s instructions. Briefly, cells were plated into a 96-well plate at a density of 5 × 10^3^ cells per well. After overnight incubation, the medium was cultured. After that, cells were incubated in the dark with CCK-8 (Beyotime Institute Biotech, China) solution for 2 h. The absorbance was measured at 450 nm with a Varioskan Flash Spectral Scanning Multimode Reader (Thermo Electron Corporation, United States).

### 2.10 Senescence-associated β-galactosidase (SA-β-gal) staining

Cells were immobilized using 4% paraformaldehyde. Subsequently, they were stained and incubated at 37°C for 24 h. To assess the proportion of senescence-associated SA-β-gal-positive cells (Mirus Bio Co., United States), cells within each dish were manually counted. Images of the stained cells were captured using a phase-contrast microscope (Olympus, Japan).

### 2.11 Determination of SOD, GSH-Px, CAT, and MDA

The activities of SOD, GSH-Px, CAT, and the level of MDA were measured using commercially available kits according to the manufacturer’s instructions. SOD activity was assessed based on its ability to inhibit the reduction of nitroblue tetrazolium. GSH-Px activity was determined by the oxidation of glutathione. CAT activity was measured via the decomposition of H2O2. MDA content was quantified using the thiobarbituric acid reactive substances method, reflecting lipid peroxidation levels.

### 2.12 Cell cycle analysis

Following UVA exposure, cells were subjected to cell cycle assays (purchased from Beyotime Institute of Biotechnology, China) at 24, 48, and 72 h. Subsequently, the cells were harvested, fixed in 70% cold ethanol, and stored at 4°C for 24 h. After that, the cells were stained with RNase at 37°C in the dark for 1 h. Finally, the cell cycle was analyzed using flow cytometry (BD FACSCalibur, United States).

### 2.13 Quantitative real-time PCR

Cellular RNA was extracted using Trizol. RNA was then used for cDNA synthesis. The reaction mixture was prepared using a SYBR Green Master Mix, and the PCR conditions were as follows: initial denaturation at 95°C for 10 min; followed by 45 cycles of denaturation at 94°C for 15 s, annealing at 59°C for 20 s, and extension at 72°C for 30 s.

Sequences of primers:

**Table:** 

Gene	Forward primer (5′–3′)	Reverse primer (5′–3′)	Product length (bp)
GAPDH	CCATGGG GAAGGTG AAGGTC	AGTGATGG CATGGA CTGTGG	448
MMP-1	AAAATTAC ACGCCAGA TTTGCC	GGTGTGACA TTACTCCA GAGTTG	82
p16	GATCCAG GTGGGTA GAAGGTC	CCCCTGCAA ACTTCGTCCT	74
p53	CAGCACA TGACGGA GGTTGT	TCATCCAA ATACTCCA CACGC	91
BCL-2	GGTGGGG TCATGT GTGTGG	CGGTTCA GGTACTCA GTCATCC	103
HSP90AA1	AGGAGGT TGAGAC GTTCGC	AGAGTTCGA TCTTGTT TGTTCGG	223

### 2.14 siRNA transfection and knockdown

Gene knockdown was performed using small interfering RNA (siRNA). A human HSP90AA1-specific siRNA (siHSP90AA1) and a non-targeting scrambled control siRNA (siNC) were designed and synthesized by GenePharma (Shanghai, China). For transfection, HSFs were seeded in plates and allowed to reach 40–50% confluence. The siRNAs were transfected into cells using Lipofectamine 3000 (Invitrogen, CA, United States) according to the manufacturer’s protocol. The sequence of the HSP90AA1 siRNA was: sense, 5′-GGUCAAGAAAUGCCUAGAATT-3′; antisense, 5′-UUCUAGGCAUUUCUUGACCTT-3′. After 6 h of transfection, the medium was replaced with fresh complete medium. Cells were then cultured for an additional 42 h (total 48 h post-transfection) to achieve maximal knockdown efficiency before being subjected to β-sitosterol treatment and UVA irradiation. Knockdown efficiency was confirmed by Western blot analysis.

### 2.15 Western blot analysis

Protein was extracted using 1% SDS lysis buffer, followed by separation of nuclear and cytoplasmic fractions using a commercial extraction kit (Beyotime, China, P0027). Protein samples were then separated by SDS–PAGE (Beyotime, China, P0012AC) and transferred onto 0.45 μm PVDF membranes (GE Healthcare, Germany). The membranes were blocked with 5% BSA for 1 h at room temperature and subsequently incubated overnight at 4 C° with the following primary antibodies: anti-HSP90AA1 (Affinity, BF0084, 1:1,000) and anti-GAPDH (Proteintech, 1:2,500). After washing, membranes were incubated with the corresponding horseradish peroxidase (HRP)-conjugated secondary antibodies (Beyotime, China) for 1 h at room temperature. Protein bands were visualized using an enhanced chemiluminescence (ECL) reagent (NCM Biotech, China, 10,100) and imaged with a ChemiDoc™ MP Imaging System (Bio-Rad, United States).

### 2.16 Statistical analysis

All quantitative experiments were performed with three independent biological replicates (*n* = 3), each replicate representing cells cultured and treated at different times. The sample size (*n* = 3) was chosen based on common practice in the field for preliminary mechanistic studies and our preliminary data, which showed consistent and reproducible effects with this number of replicates. Graphpad was used for statistical analysis. Data are presented as mean ± standard deviation (SD). The data were analyzed with ANOVA followed by an LSD test for individual comparisons between group means. Statistical analysis of *P* < 0.05 was considered significant.

## 3 Results

### 3.1 Prediction of active components in black soybean

Based on the unique characteristics of various components of drugs and their target receptors, through the absorption, distribution, metabolism, and excretion (ADME) screening of drugs, ten potential compounds were identified in black soybean (OB ≥ 30% and DL ≥ 0.18). These compounds included β-sitosterol, stigmasterol, gondoic acid, FA (fulvic acid), delphinidin, daidzein-4,7-diglucoside, pramoxine, cycloartenone, L-folinic acid, and N-(p-hydroxyphenethyl) actinidine. Their corresponding OB and DL values are provided in Table 1.

**TABLE 1:** OBand DL values of the candidate compounds identified from black soybean.

Compound name	OB (%)	DL
Cycloartenone	40.57	0.79
Stigmasterol	43.83	0.76
β-sitosterol	36.91	0.75
L-Folinic acid	31.79	0.74
FA	68.96	0.71
Daidzein-4,7-diglucoside	47.27	0.67
Delphinidin	40.63	0.28
Gondoic acid	30.7	0.2
N-(p-Hydroxyphenethyl) actinidine	62.16	0.19
Pramoxine	49.08	0.19

### 3.2 Construction and analysis of the protein-protein interaction network

Initial screening yielded 12,554 aging-related targets from disease databases. Venn analysis identified 63 overlapping targets, comparing the active target components of the disease with those in the compound formula (Figures 2A,B). These 63 intersecting targets constituted the node set for the subsequent compound-disease target network (Figure 2C). And protein-protein interaction (PPI) network (60 nodes, 308 edges; Figure 2D) revealed core targets through multidimensional topological analysis, including: BC: Betweenness Centrality (bridging capacity in shortest paths), CC: Closeness Centrality (propagation efficiency to all nodes), DC: Degree Centrality (direct connection quantity), Eigenvector (influence of neighboring hubs), LAC (local connection density), and Network-scale parameters (global topological features). Twenty key targets were prioritized for further investigation (Figure 2E; Table 2).

**TABLE 2:** Top 20 targets information of PPI network.

Name	BC	CC	DC	Eigenvector	LAC	Network
MAOA	286.98	0.57	19.00	0.17	5.89	10.31
MAOB	106.74	0.53	15.00	0.15	5.73	7.57
SLC6A2	59.16	0.51	13.00	0.10	4.92	6.60
SLC6A4	60.58	0.49	15.00	0.10	6.40	8.78
ADRA2A	85.83	0.50	13.00	0.10	4.77	5.71
DRD1	227.62	0.52	16.00	0.11	5.13	7.72
SLC6A3	159.07	0.51	16.00	0.12	5.25	7.75
PTGS1	86.07	0.50	14.00	0.15	4.86	6.89
PRKCA	90.44	0.52	15.00	0.19	6.27	7.97
PTGS2	447.55	0.61	28.00	0.31	8.79	20.74
PRKACA	365.87	0.57	20.00	0.22	5.90	9.54
CDK2	126.40	0.49	15.00	0.19	7.33	9.28
GSK3B	140.86	0.56	19.00	0.24	8.53	12.84
AR	55.22	0.48	14.00	0.19	7.71	9.38
CASP3	147.82	0.55	21.00	0.26	9.05	15.05
PGR	47.17	0.49	16.00	0.20	8.38	12.28
HSP90AA1	160.53	0.55	20.00	0.24	8.10	12.95
JUN	98.12	0.54	20.00	0.25	9.60	15.25
BCL2	102.53	0.55	20.00	0.26	9.50	14.13
CASP9	52.49	0.48	11.00	0.16	7.27	8.18

BC, Betweenness Centrality, CC, Closeness Centrality, DC, Degree Centrality, MAOA, Monoamine oxidase A, MAOB, Monoamine oxidase B, SLC6A2, Solute carrier family 6 member 2 (Norepinephrine transporter), SLC6A4, Solute carrier family 6 member 4 (Serotonin transporter), ADRA2A, Adrenoceptor alpha 2A, DRD1, Dopamine receptor D1, SLC6A3, Solute carrier family 6 member 3 (Dopamine transporter), PTGS1, Prostaglandin-endoperoxide synthase 1 (Cyclooxygenase-1), PRKCA, Protein kinase C alpha, PTGS2, Prostaglandin-endoperoxide synthase 2 (Cyclooxygenase-2), PRKACA, Protein kinase cAMP-activated catalytic subunit alpha, CDK2, Cyclin-dependent kinase 2, GSK3B, Glycogen synthase kinase 3 beta, AR, Androgen receptor, CASP3, Caspase 3, PGR, Progesterone receptor, HSP90AA1, Heat shock protein 90 alpha family class A member 1, JUN, Jun proto-oncogene (AP-1 transcription factor subunit), BCL2, B-cell lymphoma 2, CASP9, Caspase 9.

### 3.3 GO enrichment analysis and KEGG pathway analysis

As shown in Figure 2F, through Gene GO analysis, we identified 20 significantly related biological processes, cellular components, and molecular functions. These functional modules encompassed crucial biological phenomena related to aging, such as responses to steroid hormones, responses to exogenous substances, and the mitochondrial outer membrane (Figure 2F). To further clarify the molecular mechanisms by which black soybean delay aging, we conducted KEGG pathway enrichment analysis. The analysis results screened out the 20 most significant pathways, including the serotonergic synapse, hormone signaling, estrogen signaling pathway, and p53 signaling pathway (Figure 2G) (26–28). Among them, based on the analysis of the number of differential genes, the serotonergic synapse signaling pathway exhibited the most prominent enrichment characteristics (Figure 2H). Additionally, the estrogen signaling pathway also demonstrated significant enrichment, and its roles in maintaining cellular homeostasis, regulating oxidative stress, and delaying tissue degradation cannot be overlooked (Figure 2I). Notably, there exists a complex co-regulatory network among these key pathways, jointly regulating multiple key biological processes during the aging process. For example, the p53 signaling pathway regulates cell-cycle arrest and apoptosis, collaborating with the estrogen signaling pathway to maintain cellular homeostasis (35); the serotonergic synapse pathway, by modulating the neuroendocrine system, interacts with the hormone signaling pathway to jointly maintain the body’s internal environment balance (36). Simultaneously, these pathways, through regulating processes such as oxidative stress response and DNA damage repair, collaboratively delay the process of cellular aging (37). This multi-pathway synergistic mechanism provides novel insights into the in-depth understanding of the molecular mechanisms by which black soybean delay aging.

### 3.4 Machine learning

**FIGURE 3:**
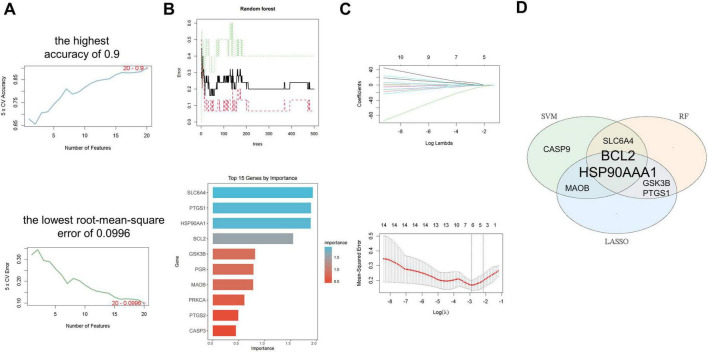
Bioinformatics-driven feature selection. **(A)** Performance evaluation of the SVM-RFE algorithm for feature (gene) selection. The plot shows the model accuracy versus the number of features retained. **(B)** Feature importance ranking plot from the RF algorithm. Genes are ranked by their Gini importance score. **(C)** Feature selection trajectory of the LASSO logistic regression model. The vertical dashed line indicates the optimal lambda value selected via 10-fold cross-validation. **(D)** Venn diagram illustrating the consensus key genes identified by the intersection of the top candidates from the SVM-RFE, RF, and LASSO algorithms, pinpointing HSP90AA1 and BCL2 as the core targets.

The top 20 targets from PPI analysis (Table 2) were used as input features for machine learning algorithms. This integration ensures biological relevance (via PPI topology) and statistical robustness (via algorithmic feature selection). Employing machine-learning methods, we screened the important key genes in the training dataset through three different machine-learning algorithms. The SVM-RFE algorithm achieved the highest accuracy of 0.9 and the lowest root-mean-square error of 0.0996 (Figure 3A). And the RF algorithm assigned an importance score to each gene, and we set a threshold of 0.6 to determine the key genes (Figure 3B). Meanwhile, the LASSO algorithm screened the initial 20 genes into 9 key genes (Figure 3C). Eventually, based on AvgRank and Importance, we selected the top five genes from SVM and RF, respectively, and intersected them with the gene set determined by LASSO to identify two core genes, HSP90AA1 and B-cell lymphoma-2 (BCL2), for subsequent research (Figure 3D).

### 3.5 Molecular docking studies

**FIGURE 4:**
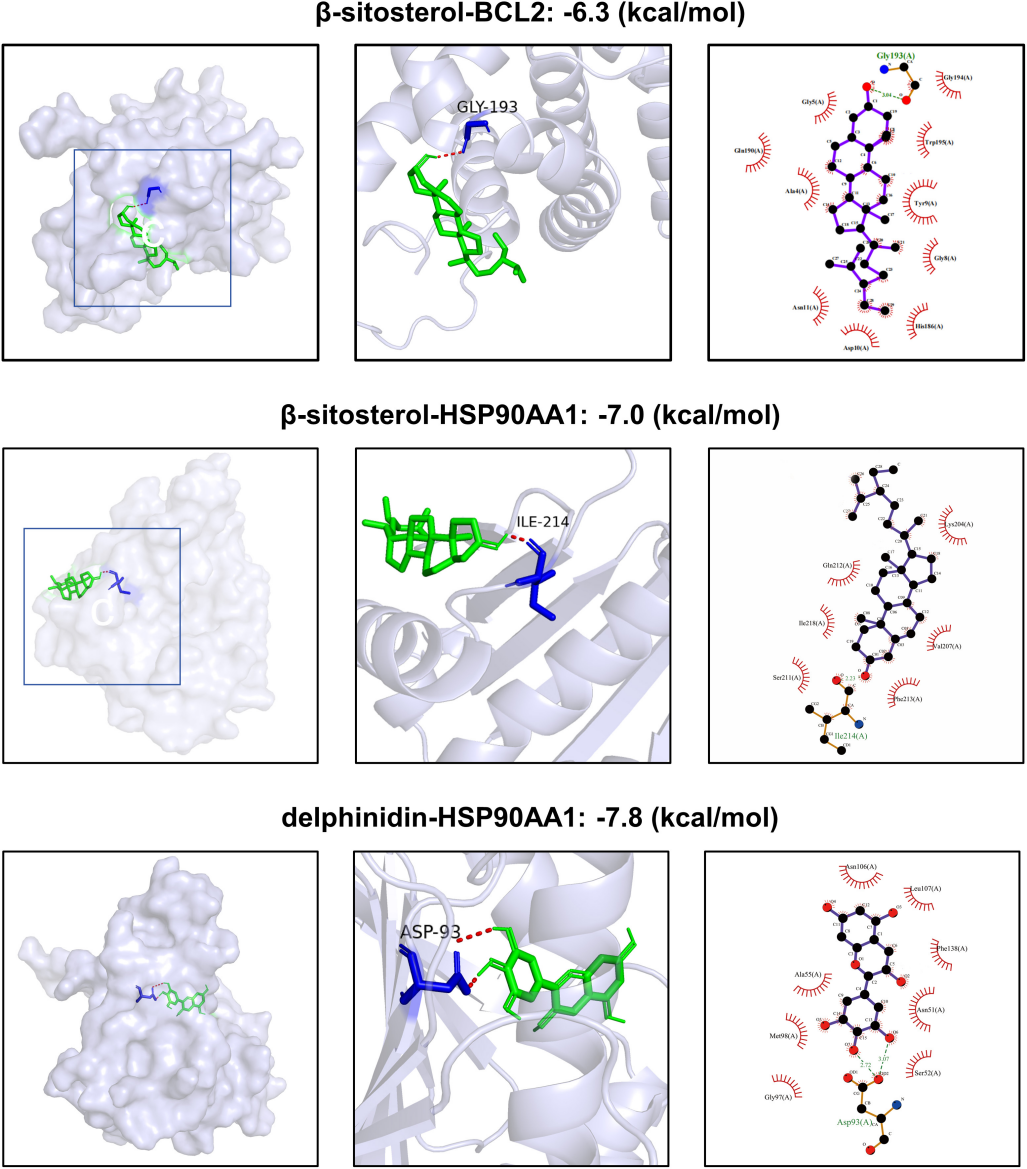
Virtual molecular docking results.

Based on the compound-target network analysis (Supplementary Table 1), HSP90AA1 was associated with β-sitosterol and delphinidin, while BCL2 was linked to β-sitosterol. Molecular docking was therefore performed exclusively on these three compound-target pairs to validate their binding potential (Figure 4). This analysis aimed to determine the crucial roles of these compounds in treating aging. Generally, a binding energy lower than −5 kcal/mol indicates a strong binding force between the ligand and the receptor (38). Our research results demonstrate that the small molecules possess good binding affinities for the proteins encoded by the core genes. Furthermore, the interactions between all ligand-macromolecule complexes are mediated by hydrogen bonds, ensuring the stability of these bindings. Notably, β-sitosterol exhibited favorable binding energies for both core targets, indicating its significant effects. Detailed analysis revealed that β-sitosterol docks into the N-terminal ATP-binding pocket of HSP90AA1, forming a stable complex primarily stabilized by hydrophobic interactions and a critical hydrogen bond between its hydroxyl group and the backbone of ILE-214 (Figure 4). This key residue is located within the 9–236 functional domain of HSP90AA1, a region annotated in UniProt for client protein interaction. Thus, the core hydrogen bond interaction centers on ILE-214 within this vital functional domain, suggesting β-sitosterol directly engages a key site to potentially modulate HSP90AA1 chaperone activity. Given its superior binding affinity to both core targets (HSP90AA1 and BCL2) and this specific interaction with the functionally crucial HSP90AA1 domain, our subsequent *in vitro* experiments were carried out using β-sitosterol.

### 3.6 β-sitosterol protects HSFs from UVA induced photoaging

**FIGURE 5:**
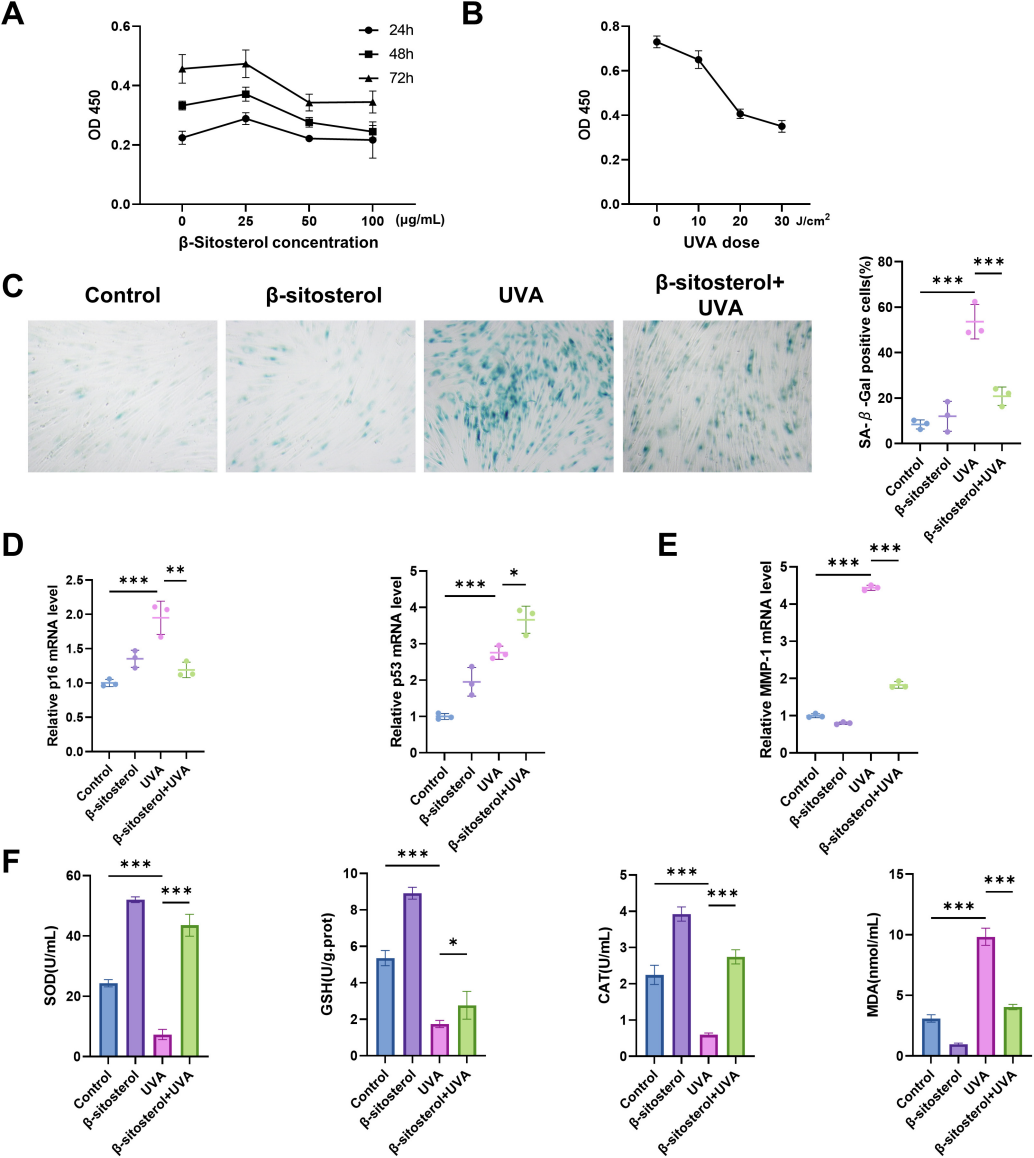
β-sitosterol attenuates UVA-induced photoaging in HSFs. **(A)** Cell viability assay determining optimal β-sitosterol concentration. **(B)** Proliferation activity of HSFs under graded UVA irradiation. **(C)** β-sitosterol-mediated suppression of UVA-induced senescence. **(D)** Analysis of p16/p53 mRNA expression. **(E)** mRNA level of MMP-1 demonstrating β-sitosterol inhibition of UVA-induced matrix degradation. **(F)** Antioxidant profiles and MDA levels under UVA exposure with/without β-sitosterol pretreatment. Data shown as mean ± SEM, *n* = 3, *p < 0.05, **p < 0.01, ***p < 0.001.

To determine the appropriate concentration of β-sitosterol, we evaluated its cytotoxicity at concentrations ranging from 0 to 100 μg/mL. The proliferation activity of HSFs cultured with β-sitosterol peaked at a concentration of 25 μg/mL (Figure 5A). Therefore, we chose 25 μg/mL for a 24 h treatment in subsequent mechanism studies. HSFs were exposed to UVA at doses of 10, 20, and 30 J/cm^2^ for 2 weeks (Figure 5B). HSFs exposed to 10 J/cm^2^ UVA exhibited minimal proliferation inhibition (10.96%), whereas irradiation at 20 and 30 J/cm^2^ induced significantly higher cytotoxicity (44.29 and 52.05%). This subtoxic dose (10 J/cm^2^) effectively simulated chronic photodamage characteristics while avoiding acute cell death, meeting established criteria for studying prolonged photoaging mechanisms. Dose-dependent responses confirmed through proliferation rates guided optimal parameter selection. Thus, we determined that 10 J/cm^2^ was the most suitable sub-cytotoxic UVA irradiation dose for further research.

Our findings further demonstrated that β-sitosterol could reduce the proportion of senescent cells. As anticipated, UVA irradiation significantly increased the number of positively stained HSFs, while β-sitosterol treatment effectively suppressed the UVA-induced expression of senescence-associated β-galactosidase (SA-β-gal) (Figure 5C).

UVA irradiation significantly induces cell cycle arrest in skin cells, a process predominantly regulated by the key molecular regulators p16 and p53. p16 inhibits the G1-to-S phase transition by suppressing CDK4/6 activity, whereas p53 mediates cell cycle arrest, DNA repair, and apoptosis through the regulation of downstream target genes, such as p21. In this study, we observed that UVA exposure markedly upregulated the expression levels of both p16 and p53 in HSFs. Intriguingly, β-sitosterol treatment significantly mitigated the UVA-induced upregulation of p16 but further enhanced p53 expression (Figure 5D). This may be attributed to the multifaceted role of p53, which not only regulates the cell cycle but also plays a crucial role in the clearance of senescent cells. β-sitosterol may maintain skin tissue homeostasis by promoting p53-mediated apoptosis of senescent cells.

We also investigated whether β-sitosterol could protect cells from UVA-induced production of MMPs. As expected, UVA irradiation elevated the MMP-1 level, while β-sitosterol protected cells from the detrimental effects of this enzyme (Figure 5E).

Long-term UVA irradiation causes cumulative damage and oxidative reactions, accelerating the photoaging process, leading to the depletion of antioxidant substances and the accumulation of oxidative products. In our study, the activities of superoxide dismutase (SOD), catalase (CAT), and glutathione peroxidase (GSH-px) in HSFs were significantly decreased in the UVA-irradiated group, while the level of malondialdehyde (MDA) was significantly increased. Meanwhile, pre-treatment with β-sitosterol significantly inhibited UVA-induced MDA production and antioxidant suppression (Figure 5F).

### 3.7 β-sitosterol protects HSFs from photoaging via HSP90AA1

**FIGURE 6:**
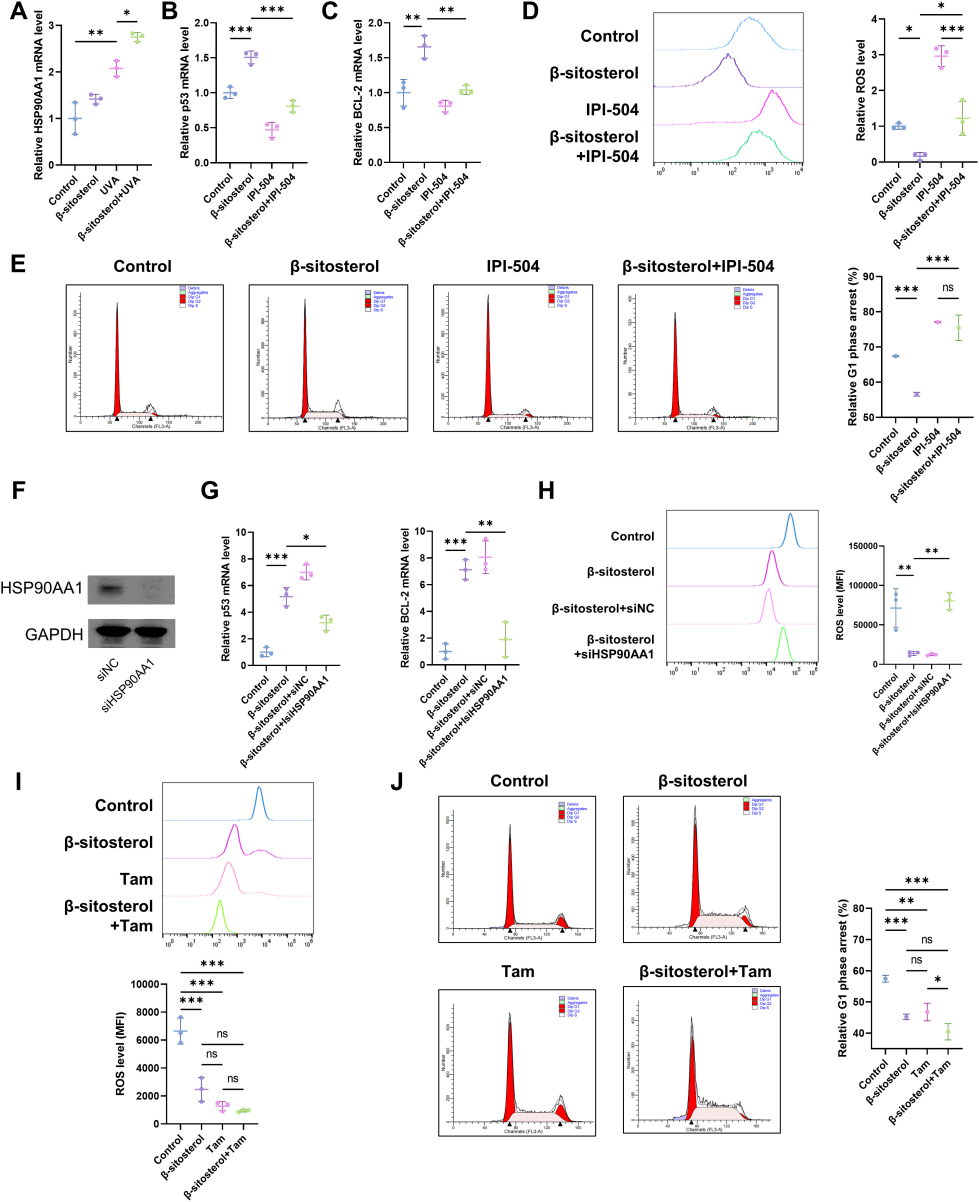
HSP90AA1 mediates β-sitosterol’s anti-photoaging effects. **(A)** HSP90AA1 expression upregulated by UVA and further enhanced by β-sitosterol (mRNA). **(B,C)** Co-treatment with HSP90AA1 inhibitor IPI-504 reverses β-sitosterol-induced stabilization of pro-survival BCL-2 and p53 (mRNA). **(D)** ROS scavenging capacity of β-sitosterol blocked by IPI-504 (DCFH-DA assay). **(E)** Cell cycle analysis showing β-sitosterol alleviates UVA-induced G1 arrest, abrogated upon HSP90AA1 inhibition. **(F)** Western blot analysis confirming efficient knockdown of HSP90AA1 protein by specific siRNA (siHSP90AA1). **(G)** HSP90AA1 knockdown abolishes β-sitosterol’s stabilizing effect on p53 and BCL-2 mRNA levels under UVA stress. **(H)** HSP90AA1 knockdown blocks the ability of β-sitosterol to scavenge UVA-induced ROS. **(I)** ROS scavenging capacity of Tam (1 μmol/L). **(J)** Cell cycle analysis showing Tam alleviates UVA-induced G1 arrest. Data shown as mean ± SEM, *n* = 3, *p < 0.05, **p < 0.01, ***p < 0.001.

HSP90AA1, a crucial molecular chaperone protein, plays an indispensable role in cellular stress responses and the maintenance of protein homeostasis (39). We measured the expression levels of HSP90AA1. The results showed that UVA irradiation significantly upregulated the expression of HSP90AA1 (Figure 6A). This phenomenon is presumably an indication of the activation of a protective stress-response mechanism in cells to counteract the damage caused by UVA irradiation. Notably, β-sitosterol could further enhance the expression of HSP90AA1, suggesting that β-sitosterol may be involved in regulating the process of cellular photoaging through HSP90AA1.

To further explore the core role of HSP90AA1 in this process, we treated HSFs with its specific inhibitor, IPI-504, and conducted experiments in combination with UVA irradiation. The results revealed that β-sitosterol could stabilize the expression levels of p53 and BCL-2, two genes that play key roles in the regulation of cell apoptosis and survival (Figures 6B,C). The treatment with IPI-504 reversed this effect of β-sitosterol, indicating that the inhibition of HSP90AA1 disrupts the balance between apoptosis and survival within cells, leading to an increase in normal cell apoptosis and a decrease in the clearance of senescent cells. In terms of oxidative stress and the cell cycle, β-sitosterol demonstrated significant protective effects (Figures 6D,E). It could significantly reduce the level of reactive oxygen species (ROS), a marker of oxidative stress, thereby alleviating intracellular oxidative damage. Meanwhile, it could also relieve the UVA-induced G1 phase cell-cycle arrest, ensuring the normal progression of the cell cycle. Nevertheless, when HSP90AA1 was inhibited, the above-mentioned protective effects of β-sitosterol were blocked, further confirming the central role of HSP90AA1 in the process by which β-sitosterol exerts its protective effects. To provide genetic evidence complementing the pharmacological inhibition, we performed siRNA-mediated knockdown of HSP90AA1. Transfection with HSP90AA1-specific siRNA (siHSP90AA1), but not a scrambled control siRNA (siNC), efficiently reduced HSP90AA1 protein levels (Figure 6F). Under this condition, the stabilizing effects of β-sitosterol on UVA-induced p53 and BCL-2 expression were abolished (Figure 6G). Consistent with this, the ability of β-sitosterol to scavenge UVA-induced ROS was also blocked in HSP90AA1-knockdown cells (Figure 6H). We also tested Tamoxifen (Tam), a reported HSP90 ATPase enhancer known to reduce oxidative damage in photoaging models (40, 41). Both β-sitosterol and Tam alleviated UV-induced ROS accumulation and cell cycle arrest. The combined treatment showed superior efficacy in alleviating cell cycle arrest compared to Tam monotherapy, which suggests β-sitosterol primarily exerts its protective effects through HSP90 pathway activation, whereas its enhanced combinatorial benefits in cell cycle regulation may involve additional biological targets (Figures 6I,J).

## 4 Discussion

Through integrative network pharmacology and experimental validation, we elucidated the molecular basis of black soybean’s anti-photoaging effects. Machine learning convergence on HSP90AA1 highlights its pivotal role within the aging-related network we constructed. β-sitosterol emerged as the core bioactive component based on target spectrum and binding affinity.

Among the active components identified in black soybean, β-sitosterol was prioritized for experimental validation. This selection was based on network pharmacology and molecular docking analyses which revealed that β-sitosterol was associated with both core targets (HSP90AA1 and BCL2) identified by machine learning and exhibited high binding affinity to HSP90AA1 (Figure 4). While β-sitosterol has been reported to induce p53 in cervical carcinoma cells and modulate BCL2 in inflammation models, and HSP90 inhibition is known to destabilize client proteins including p53 and BCL2 in oncology, our findings delineate a novel function in skin photoaging (42). Here, we demonstrate that β-sitosterol, a core component of black soybean, upregulates HSP90AA1 activity, leading to the concurrent stabilization of both p53 and BCL2 and promoting cell survival under stress. This mechanism was elucidated through our integrated computational-experimental strategy. It is important to note that black soybean contains other bioactive components, and our initial network pharmacology analysis revealed multiple potential targets interconnected within the aging-related PPI network (Figures 2D,E; Table 2). The β-sitosterol-HSP90AA1 axis we validated here thus represents a significant, but likely not exclusive, pathway through which black soybean exerts its anti-aging effects. Future studies investigating the roles of other components and their synergistic interactions will provide a more comprehensive understanding of this medicinal-edible substance.

PPI network analysis indicated that the anti-aging target groups of black soybean exhibited remarkable modular characteristics. HSP90AA1, as one of the core hubs, formed a tight functional cluster with nodes such as BCL2, CASP3, GSK3B, AR, and PGR (Figures 2D,E). This structure suggests that the active components of black soybean may exert a synergistic anti-aging effect by intervening in functions such as protein homeostasis (HSP90AA1), apoptosis regulation (BCL2/CASP3), glucose metabolism and oxidative stress balance (GSK3B), and hormone receptor signal transduction (AR/PGR) (43–45). Integrated functional analysis combining PPI and KEGG pathway data revealed that HSP90AA1 engages concurrently with both the serotonergic-CASP3 axis and estrogen-BCL2 axis, connecting the serotonergic and estrogen signaling pathways. Mechanistically, HSP90AA1 operates at this pathway intersection through two interdependent mechanisms: modulating ERα stability to maintain BCL2-mediated survival signaling while interacting with CASP3 to regulate stress-responsive senescence signals. Experimental inhibition of HSP90AA1 disrupted cellular homeostasis mediated by apoptosis-associated and oxidative stress-related factors, confirming its essential role in synchronizing hormonal survival pathways with neurotransmitter-mediated stress adaptation. This regulatory capability resonates with traditional Chinese medicine’s “holistic view,” wherein a single molecular hub (HSP90AA1) coordinates divergent biological processes encompassing hormonal signaling and neuroendocrine stress responses toward unified therapeutic effects. The multi-level regulatory pattern also highly aligns with the characteristics of systemic imbalance during the aging process, providing a new perspective for understanding the systematic pharmacological effects of natural products.

Molecular docking revealed that β-sitosterol exhibited a strongly negative binding energy with HSP90AA1 (−7.0 kcal/mol), indicating a high-affinity interaction. This finding has dual significance. On the one hand, as the central processor of cell signal transduction, the stabilization of HSP90AA1 may enhance the functional conformations of client proteins such as p53 and BCL2. This function is consistent with its topological position as a highly connected hub in our PPI interaction network (Figure 2E), where it likely serves as a coordinator integrating survival (BCL2) and stress-response (p53) signals. On the other hand, the allosteric regulation of HSP90AA1 by β-sitosterol may reshape its interaction pattern with co-chaperones (such as CDC37), thereby selectively regulating specific signal branches (46). More importantly, as a phytosterol, β-sitosterol also has a structure similar to that of human cholesterol. It may indirectly affect the signal transduction efficiency by competitively binding to membrane receptors or modifying the lipid raft structure (47). This characteristic of structural mimicry and functional regulation provides a molecular basis for explaining the advantage of gentle systemic regulation exhibited by food-medicine homologous substances.

*In vitro* experiments further supported this hypothesis. Under UVA exposure, β-sitosterol demonstrated distinct bidirectional regulatory effects: it suppressed the p16-mediated SASP while promoting p53-dependent clearance of senescent cells (Figure 5D). This seemingly contradictory phenomenon may be attributed to the functional polymorphism of p53. In the initial stage of DNA damage, p53 induces cell-cycle arrest through p21 to allow for repair. When the damage is irreversible, it initiates the apoptosis program mediated by the BAX/BCL2 axis. The upregulation of p53 by β-sitosterol may achieve precise “repair and clearance” management through temporal regulation, as evidenced by prior studies showing that β-sitosterol-treated Hela cells exhibit significantly elevated p53 mRNA levels, which correlates with its pro-apoptotic effects in cancer models (42). In addition, its synergistic effect on HSP90AA1 suggests that the dynamic balance of the HSP90-p53-BCL2 complex may be a key switch determining cell fate. This regulatory mechanism likely enables β-sitosterol to simultaneously activate p53-dependent DNA repair (via NER/BER pathways) and promote apoptotic clearance of damaged cells (48). Such coordinated actions may drive the observed reduction in UV-induced collagen degradation (Figure 5E), establishing β-sitosterol as a multifunctional anti-photoaging compound.

The hub position of HSP90AA1 was verified at multiple levels in this study. As the protein quality control center, the upregulation of its expression not only enhances the refolding ability of misfolded proteins but also affects the downstream signal network by regulating the stability of client proteins such as p53 and AKT. Notably, β-sitosterol-induced HSP90AA1 upregulation (Figure 6A) may functionally compensate for exogenous activation in the context of cellular stress. This ligand-mediated enhancement could represent a mechanism for augmenting stress-responsive pathways. When HSP90AA1 was inhibited by IPI-504 (Figures 6B–E), the antioxidant and anti-apoptotic effects of β-sitosterol were significantly weakened, confirming that HSP90AA1 is a key mediator for its function. Notably, the synergistic effect between HSP90AA1 and BCL2 may constitute a “double-insurance” mechanism. HSP90AA1 inhibits the apoptosis pathway by maintaining the stability of BCL2 and simultaneously balances the pro-survival/pro-apoptosis signals by regulating the nuclear translocation of p53. This two-way regulatory network provides a new framework for explaining the cytoprotective effect of β-sitosterol under oxidative stress.

The anti-photoaging mechanism mediated by the HSP90AA1-p53/BCL2 axis, as elucidated in this study, positions β-sitosterol as a promising candidate for skin health applications. Its potential is further supported by established evidence of diverse skin benefits. Independent research has demonstrated the efficacy of β-sitosterol in ameliorating atopic dermatitis-like lesions by downregulating thymic stromal lymphopoietin via inhibition of the Caspase-1/NF-κB pathway in mast cells, underscoring its anti-inflammatory properties (49). Furthermore, β-sitosterol enhances skin barrier function and hydration by upregulating aquaporin-3 and cornified envelope proteins in keratinocytes, and promotes hyaluronic acid synthesis in fibroblasts (50). These complementary actions converge with our findings on oxidative stress mitigation and senescence regulation, portraying β-sitosterol as a multifunctional skin-protective agent. To translate these cellular and molecular insights into effective topical therapies, addressing its inherent physicochemical challenges such as poor aqueous solubility is essential. Promisingly, advanced delivery strategies including encapsulation in nanocarriers like cubosomes or integration into dissolving microneedle systems have been successfully employed to significantly enhance the dermal bioavailability and efficacy of β-sitosterol, as evidenced in models of alopecia (51). The exploration and optimization of these delivery platforms, particularly biocompatible lipid-based systems like liposomes, represent a crucial future direction for developing potent and stable β-sitosterol-based formulations against skin aging.

While this study offers novel insights into the anti-photoaging mechanisms of black soybean, several limitations should be acknowledged. Regarding the computational screening strategy, the machine learning-based target prioritization relied on a single skin aging transcriptomic dataset (GSE75192). Although the convergence of three distinct algorithms and subsequent experimental validation mitigate bias concerns, incorporating multiple independent datasets in future studies would strengthen the generalizability of bioinformatic predictions. On the experimental and mechanistic level, our conclusions are primarily based on a single HSF cell model under UVA-induced stress. This model, while informative, does not fully recapitulate the complexity of the skin tissue microenvironment *in vivo*. Moreover, the potential contributions of other black soybean components, such as delphinidin which also showed binding to HSP90AA1 *in silico*, to the overall anti-aging effect were not experimentally isolated, leaving open the possibility of synergistic interactions within the phytochemical ensemble. Furthermore, the broader multi-target potential suggested by our network pharmacology analysis, including other highly ranked nodes like AKT, remains to be functionally explored. To advance these findings, future research should prioritize efficacy validation in established animal models of photoaging to bridge the gap between cellular mechanisms and whole-organism physiology. Additionally, integrating multiple omics datasets will help construct a more robust and generalizable anti-aging target network. Finally, systematic investigation of multi-component interactions through techniques like molecular dynamics simulation will be crucial to elucidate the systems-level pharmacology of black soybean.

## 5 Conclusion

Our study reveals the mechanism of black soybean in delaying skin photoaging from a systems biology perspective. The core component β-sitosterol targets the HSP90AA1 hub node, dynamically regulates the activity of the p53-BCL2 signaling axis, and synergistically inhibits oxidative stress, matrix degradation, and the accumulation of senescent cells. These findings not only provide molecular evidence for the medicinal-edible value of black soybean but also offer a theoretical basis for the application of black soybean in the development of functional skin care products and health products. Moreover, it suggests that targeting the HSP90AA1-related pathway may become a new direction in anti-aging research.

## Data Availability

The original contributions presented in the study are included in the article/supplementary material, further inquiries can be directed to the corresponding authors.
