# Supplementary material for: Network pharmacology, machine learning, and experiments uncover β-sitosterol targeting HSP90AA1 in medicinal-edible black soybean against aging
Source: Front Med (Lausanne). 2026 Feb 16;13:1749856. doi: 10.3389/fmed.2026.1749856 (PMC12950797; doi:10.3389/fmed.2026.1749856)
Supplement: Supplementary file 1 [file Table_1.docx]

**Supplementary Table1：Detailed machine learning outputs**

| **SVM** | **FeatureName** | **FeatureID** | **AvgRank** |
| --- | --- | --- | --- |
| 1 | BCL2 | 5 | 2.8 |
| 2 | HSP90AA1 | 4 | 3.2 |
| 3 | MAOB | 2 | 4.6 |
| 4 | CASP9 | 7 | 6 |
| 5 | SLC6A4 | 12 | 6.6 |
| **RF** | **Gene** | **importance** | |
| 1 | SLC6A4 | 1.92650797875711 | |
| 2 | PTGS1 | 1.89185807656396 | |
| 3 | HSP90AA1 | 1.88480809867807 | |
| 4 | BCL2 | 1.54732593839314 | |
| 5 | GSK3B | 0.813783160322953 | |
| **LASSO** | **Gene** | **Coef** | |
| 1 | BCL2 | -10.35325816 | |
| 2 | MAOB | 6.07823025365097 | |
| 3 | HSP90AA1 | -2.107988349 | |
| 4 | GSK3B | 1.23679905236836 | |
| 5 | PTGS1 | -1.474906264 | |
